# Electroacupuncture and P2X receptors: purinergic mechanisms in pain modulation: A narrative review with structured evidence mapping

**DOI:** 10.1007/s11302-026-10184-0

**Published:** 2026-09-09

**Authors:** Rajaa Shaheen

**Affiliations:** 1 Department of Biological Sciences, University of East Anglia, Norwich, UK; 2 Faculty of Medicine and Health Sciences, An-Najah National University, Nablus, Palestine

**Keywords:** Electroacupuncture, Purinergic signaling, P2X receptors, Adenosine, Pain modulation, Acupuncture

## Abstract

Acupuncture and electroacupuncture (EA) are used for pain modulation, but the molecular events linking needling to analgesia remain incompletely defined. Purinergic signaling provides a biologically coherent framework because mechanical stimulation at acupoints releases extracellular adenosine triphosphate (ATP), which can activate P2X receptors and is subsequently metabolized into adenosine. Importantly, the receptor-level literature synthesized here is dominated by EA studies, whereas key local ATP–adenosine evidence derives from manual or traditional acupuncture. This review synthesizes evidence on purinergic mechanisms involved in acupuncture-related analgesia, with primary emphasis on EA modulation of P2X3, P2X4, and P2X7 receptors across dorsal root ganglion (DRG), spinal dorsal horn, and supraspinal cortical compartments, while distinguishing manual/traditional acupuncture evidence at the acupoint level. A narrative review with structured evidence mapping was conducted. The synthesis prioritizes mechanistic studies of manual/traditional acupuncture and EA in inflammatory, neuropathic, diabetic neuropathic, visceral, and cancer pain models, and integrates the limited available human data on acupoint adenosine signaling. Evidence is interpreted according to stimulation modality, receptor subtype, and anatomical compartment. The best-supported local pathway is the ATP–adenosine cascade: manual or traditional needling induces ATP release and CD39/CD73-mediated conversion to adenosine, activating anti-nociceptive adenosine A1 receptors (A1R). Most receptor-specific evidence, however, derives from EA. P2X3 receptors in nociceptive DRG neurons are the most consistently reproduced EA-associated target, with reduced receptor expression, membrane trafficking, and ATP-evoked currents. P2X4R-related evidence links EA analgesia with reduced spinal microglial activation, BDNF signaling, and central sensitization, but direct receptor-specific causal confirmation remains limited. P2X7R findings are compartment-specific and must be interpreted alongside EA stimulation parameters and the receptor’s requirement for relatively high or sustained extracellular ATP exposure. Purinergic signaling offers a compelling mechanistic bridge between needling-based stimulation and pain relief. The receptor-level evidence is predominantly preclinical, male-biased, and EA-centred; manual acupuncture and EA should not be treated as mechanistically interchangeable. Human receptor-level studies, sex-balanced designs, standardized reporting of EA parameters, and direct causal interrogation of receptor function are needed before P2X modulation can be considered clinically validated.

## Introduction

Chronic pain is increasingly understood not as a single disease entity but as a persistent neurobiological condition involving dynamic interactions among peripheral nociceptors, spinal glial cells, dorsal horn neurons, and supraspinal pain-modulatory networks [1, 2]. This multidimensional pathophysiology helps explain why many patients require multimodal pain-management strategies rather than reliance on a single pharmacological intervention [1]. Acupuncture and electroacupuncture (EA) are increasingly investigated as components of multimodal pain care, but their wider acceptance in biomedical practice depends on whether their analgesic mechanisms can be explained through experimentally testable and biologically plausible pathways [3, 4]. Although acupuncture is often used as an overarching clinical term, the receptor-level evidence synthesized in this review is dominated by EA studies. Manual or traditional acupuncture contributes particularly to the local ATP–adenosine–A1R literature, whereas most mechanistic evidence involving P2X3R, P2X4R, and P2X7R derives from EA models; these modalities are therefore distinguished throughout rather than treated as mechanistically equivalent.

Purinergic signaling has emerged as one of the most credible mechanistic frameworks linking acupuncture stimulation to analgesia [3, 5, 6]. Mechanical stimulation at acupoints can induce extracellular adenosine triphosphate (ATP) release from local tissue cells, including keratinocytes, mast cells, and fibroblasts [5, 7–9]. Extracellular ATP may initially activate P2X receptors—a family of ATP-gated ion channels involved in nociceptive transmission, neuronal excitability, and neuroimmune signaling [2, 10]—before being rapidly degraded by ectonucleotidases, particularly CD39 and CD73, into adenosine. Adenosine subsequently activates adenosine A1 receptors (A1R) on primary afferent terminals and contributes to local anti-nociception [5, 9, 11, 12].

Among P2X receptor subtypes, P2X3, P2X4, and P2X7 appear most relevant to acupuncture- and EA-mediated analgesia [3, 4, 6]. P2X3 receptors are predominantly expressed in small- and medium-diameter nociceptive DRG neurons and are strongly implicated in peripheral sensitization and ATP-evoked nociceptive signaling [13–15]. P2X4 receptors are important in spinal microglia, where their upregulation contributes to BDNF-mediated central sensitization and neuropathic pain signaling [16–18]. P2X7 receptors are expressed in glial populations, including satellite glial cells and astrocytes, and their role is more complex: P2X7R activity may either promote inflammatory nociceptive signaling or support supraspinal analgesic processing depending on anatomical compartment and disease context [19–22].

This review converts a mechanistically heterogeneous literature into a coherent synthesis. Its central argument is that needling-based analgesia can be understood as a multi-level purinergic process: an acupoint ATP–adenosine trigger, supported substantially by manual/traditional acupuncture evidence, is followed by receptor-subtype-specific modulation across peripheral, spinal, and supraspinal pathways that has been investigated predominantly using EA. Accordingly, the synthesis emphasizes EA in its receptor-level interpretation while retaining modality-specific manual acupuncture evidence where directly relevant.

## Review design and search strategy

The most appropriate design for this paper is a narrative review with structured evidence mapping. A formal systematic review or meta-analysis would not be suitable because the available literature is mechanistically heterogeneous, dominated by animal experiments, and varies substantially in pain model, stimulation frequency, acupoint selection, receptor assay, and outcome measure. A purely descriptive narrative review, however, would risk becoming unfocused. The present design therefore combines narrative synthesis with an explicit receptor- and compartment-based evidence map [23, 24].

The evidence was organized around five predefined domains: (1) ATP release and adenosine formation at acupoints; (2) P2X3 receptors in peripheral nociceptive neurons; (3) P2X4 receptors and spinal microglial mechanisms; (4) P2X7 receptors in satellite glial cells, spinal astrocytes, and cortical astrocytes; and (5) disease-model-specific evidence across inflammatory, neuropathic, diabetic neuropathic, visceral, and oncological pain. Searches were conducted through the Consensus academic search platform using combinations of the following terms: acupuncture, manual acupuncture, electroacupuncture, P2X3, P2X4, P2X7, purinergic receptor, ATP, adenosine, dorsal root ganglion, spinal cord, satellite glial cell, astrocyte, microglia, neuropathic pain, inflammatory pain, visceral pain, and analgesia. Because this is a narrative review rather than a systematic review, the search was used to identify and organize mechanistically relevant literature rather than to claim exhaustive retrieval.

Studies were prioritized when they directly connected manual/traditional acupuncture or EA to purinergic signaling, measured receptor expression or function, used receptor-level pharmacological or genetic manipulation, or provided translational evidence in human acupoint tissue. Stimulation modality was recorded and interpreted explicitly wherever reported. Reviews were used to support background interpretation and to identify conceptual gaps, whereas mechanistic conclusions were drawn primarily from experimental studies. Evidence synthesis is presented in two complementary formats: a study-level summary of representative major mechanistic investigations (Table 1) and a receptor-level evidence map (Table 2).

**Table 1:** Summary of representative major mechanistic studies linking acupuncture/electroacupuncture to purinergic signaling

Study	Model/Context	Acupuncture/EA Method	Main Receptor or Pathway	Key Mechanistic Finding	Main Limitation
Goldman et al. [5] (2010)	Mouse inflammatory pain model	Manual acupuncture at ST36	ATP–adenosine–A1R pathway	Needling promoted local ATP release and adenosine accumulation; A1R deletion abolished acupuncture analgesia	Animal model; downstream P2X receptor involvement not fully profiled
Takano et al. [11] (2012)	Human volunteers	Traditional acupuncture at ST36	Adenosine signaling	Human microdialysis showed increased local adenosine during acupuncture	Small human mechanistic study; no P2X receptor profiling
Tu et al. [13] (2012)	Rat CCI model	EA	P2X3R in DRG neurons	EA reduced ATP-evoked P2X3R currents in DRG neurons, providing functional electrophysiological evidence	Preclinical model; limited direct translation to humans
Chen et al. [16] (2014)	Rat CCI model	EA at ST36	IFN-γ–P2X4R microglial pathway	EA suppressed IFN-γ-driven P2X4R upregulation in spinal microglia and reduced allodynia	Rodent neuropathic pain model; no human spinal confirmation
Zhou et al. [15] (2018)	Rat diabetic neuropathic pain	EA	PKC–P2X3R membrane trafficking	EA suppressed PKC-dependent P2X3R membrane trafficking in DRG neurons	Single disease model; frequency-specific findings need broader validation
Xiang et al. [14] (2019)	CFA-induced inflammatory pain in rats	100 Hz EA at ST36 and SP6	P2X3R	EA reduced P2X3R expression in DRG and dorsal horn tissue and attenuated hyperalgesia	Single EA frequency; preclinical evidence only
Zheng et al. [17] (2020)	Spinal dorsal horn mechanism	EA	P2X4R microglial pathway	EA modulation of the P2X4R pathway reduced excitatory activity in substantia gelatinosa neurons	Mechanistic rodent evidence; clinical relevance remains indirect
Weng et al. [20] (2022)	IBS-like visceral hypersensitivity in rats	EA at abdominal acupoints	Spinal astrocytic P2X7R–NMDA-R pathway	EA inhibited spinal astrocytic P2X7R signaling and reduced NMDA-R-mediated central sensitization	Visceral pain model; limited generalizability to other pain phenotypes
Tian et al. [28] (2022)	Rat bone cancer pain model	EA	P2X3R in DRG neurons	EA reduced P2X3R expression and membrane trafficking in DRG neurons	Cancer pain evidence limited; P2X4R/P2X7R mechanisms not fully mapped
Qu et al. [18] (2023)	Rat diabetic neuropathic pain	EA	Spinal microglial P2X4R	EA suppressed P2X4R and downstream BDNF, IL-1β, and TNF-α signaling	Preclinical model; human validation absent
Lv et al. [33] (2024)	IBS-like visceral pain in rats	EA	DRG P2X7R–P2Y1R–P2X3R cascade	EA suppressed a visceral pain-related purinergic receptor cascade in DRG	May be visceral-pain-specific; broader applicability uncertain
Zhao et al. [21] (2024)	CFA-induced inflammatory pain in rats	EA	Cortical astrocytic P2X7R in RSC	P2X7R activation in RSC astrocytes supported EA analgesia	Single supraspinal study; needs replication
Wu et al. [22] (2025)	CFA-induced inflammatory pain in rats	EA	SGC P2X7R–p38 MAPK–TNF-α pathway	AAV-mediated SGC P2X7R knockdown showed that this pathway contributes to EA analgesia	Recent study; replication and human validation needed
Liang et al. [32] (2019)	Rat spinal nerve ligation model	EA	P2X3R in DRG neurons	EA was associated with reduced P2X3R expression in DRG after spinal nerve ligation, extending the P2X3R pattern to another neuropathic pain model	Preclinical model; receptor-expression evidence does not establish human translation
Hu et al. [39] (2022)	Rat diabetic hyperalgesia model	EA	DRG P2X4R and P2X7R	EA was associated with downregulation of diabetes-related P2X4R and P2X7R changes in DRG, supporting broader purinergic modulation beyond P2X3R	Preclinical model; combined receptor findings and protocol specificity limit generalization

This table summarizes representative major mechanistic and translational studies linking manual/traditional acupuncture or electroacupuncture to purinergic signaling pathways. It is not intended as an exhaustive inventory of every study cited in the narrative. Evidence is organized by pain model, stimulation method, receptor subtype or pathway, key mechanistic finding, and main limitation. *EA* Electroacupuncture, *DRG* Dorsal root ganglion, *A1R* Adenosine A1 receptor, *CFA* Complete Freund’s adjuvant, *IBS* Irritable bowel syndrome, *RSC* Retrosplenial cortex, *SGC* Satellite glial cell, *AAV* Adeno-associated virus, *CCI* Chronic constriction injury, *SNL* Spinal nerve ligation, *PKC* Protein kinase C, *IFN-γ* Interferon-gamma, *NMDA-R* N-methyl-D-aspartate receptor

**Table 2:** Evidence map comparing P2X3R, P2X4R, P2X7R, and ATP–Adenosine–A1R pathways

Receptor/Site	Main Mechanism	Role of EA	Evidence Strength
P2X3R – DRG nociceptive neurons	Peripheral sensitization through ATP-evoked neuronal excitability and increased receptor expression or membrane trafficking	Downregulates P2X3R expression/function and reduces afferent nociceptive input	Moderate–high: replicated across inflammatory, neuropathic, diabetic, visceral, and cancer pain models; functional current data available
P2X4R – spinal microglia	IFN-γ-driven microglial activation leading to BDNF release and central sensitization	Associated with reduced P2X4R upregulation, BDNF signaling, and spinal excitatory drive	Moderate associative mechanistic evidence: consistent rodent findings; direct receptor-specific causal confirmation remains limited
P2X7R – DRG satellite glial cells	Glial inflammatory signaling through p38 MAPK/TNF-α and glial–neuronal cross-talk	Inhibits pathological P2X7R inflammatory signaling; visceral cascade may involve P2X7R → P2Y1R → P2X3R	Moderate: recent cell-targeted evidence; directionality remains context-dependent; interpretation is sensitive to model and EA protocol
P2X7R – spinal astrocytes	Astrocytic P2X7R contributes to NMDA-R-mediated central sensitization and synaptic remodeling	Suppresses astrocytic P2X7R/NMDA-R pathway	Moderate: demonstrated in visceral and neuropathic pain models; interpretation is sensitive to model and EA protocol
P2X7R – cortical astrocytes (RSC)	Cortical astrocytic P2X7R activation may participate in supraspinal analgesic processing	Activation appears to support EA analgesia	Low–moderate: important but currently based on limited supraspinal evidence; requires replication; interpretation is sensitive to model and EA protocol
ATP–adenosine–A1R at acupoint	Needling-induced ATP release followed by CD39/CD73-mediated adenosine formation and A1R activation	Initiates local anti-nociception and anchors the purinergic model	High for ATP/adenosine cascade: supported by mouse and human acupoint evidence

Evidence strength was interpreted narratively based on consistency across models, availability of receptor-level or functional evidence, translational relevance, and presence or absence of human confirmation. *EA* Electroacupuncture, *A1R* Adenosine A1 receptor, *BDNF* Brain-derived neurotrophic factor, *DRG* Dorsal root ganglion, *IFN-γ* Interferon-gamma, *NMDA-R* N-methyl-D-aspartate receptor, *RSC* Retrosplenial cortex, *SGC* Satellite glial cell, *TNF-α* Tumour necrosis factor-alpha

## Acupoint purinergic signaling: from needle stimulation to adenosine A1R analgesia

The acupoint represents the primary local entry point for purinergic mechanisms in needling-induced analgesia because needle insertion and manipulation directly stimulate skin, connective tissue, local immune cells, and sensory nerve terminals [3, 5]. The intervention modality is important. Goldman et al. [5] used manual acupuncture with repeated needle rotation at ST36, providing a direct manual-acupuncture model of local purinergic analgesia. By contrast, Yao et al. [7] developed a mechanotransduction model of mast cell–nerve interaction rather than testing a clinical-style acupuncture analgesic protocol, whereas Wang et al. [8] investigated mast-cell activation at acupuncture points in an experimental acupuncture context. More recent acupoint studies have also used defined needling paradigms: Li et al. [12] administered 20-min needling at ST36 to examine keratinocyte Piezo1/CD39-dependent ATP mobilization, and Zheng et al. [25] investigated mechanically induced TRPV4-dependent extracellular ATP accumulation at the acupoint in arthritic rats. These studies support acupoint mechanotransduction but should not be assumed to represent the same stimulation modality or dose as EA receptor studies. Fibroblast evidence from Goldman et al. [26], moreover, concerns purine receptor-mediated cytoskeletal remodeling in human fibroblasts and provides general mechanistic support rather than a direct acupuncture intervention.

Extracellular ATP released at the acupoint does not remain biologically static but is rapidly shaped by local enzymatic metabolism and receptor availability [5, 9]. In the interstitial space, ATP is hydrolysed by ectonucleotidases, with CD39 contributing to ATP-to-AMP conversion and CD73 converting AMP into adenosine [9, 12]. The resulting adenosine activates A1R on nearby primary afferent terminals, thereby reducing nociceptive signaling and contributing to local anti-nociception [5, 11]. Goldman et al. [5] provided causal preclinical evidence using manual acupuncture, showing local adenosine accumulation and loss of acupuncture-induced analgesia after A1R deletion. Takano et al. [11] extended this pathway to humans using traditional acupuncture at ST36 and microdialysis measurement of local adenosine. Li et al. [9] examined CD39 activity at treated acupoints in arthritic rats, further supporting enzymatic control of extracellular nucleotide metabolism, but this evidence should be interpreted according to the specific needling protocol reported in that study rather than generalized to all acupuncture modalities.

The ATP–adenosine cascade is therefore one of the most translationally anchored components of the purinergic model, having support from manual/traditional acupuncture in both animal and human acupoint studies [5, 11]. The evidence base is not modality-uniform: key causal and human translational studies used manual or traditional acupuncture, whereas some newer acupoint mechanistic studies used other defined needling paradigms and the broader receptor-level literature is predominantly EA. The biological effect of ATP also depends on timing, concentration, receptor distribution, and enzymatic conversion into adenosine [2, 3, 10]. At the acupoint, ATP may act as a short-lived signal that is subsequently converted into a more sustained A1R-mediated anti-nociceptive response [5, 9, 12]. Pharmacological evidence from Cui et al. [27] specifically concerned EA: caffeine-mediated interference with A1R signaling impaired EA analgesia in inflammatory pain models. These modality differences are related mechanistically but should not be treated as interchangeable.

## P2X3 receptors: the most consistent peripheral target of EA

Among all P2X receptor subtypes discussed in acupuncture analgesia, P2X3 receptors currently show the strongest and most reproducible relationship with EA-mediated analgesia [6, 13–15]. P2X3 receptors are predominantly expressed in nociceptive DRG neurons, where extracellular ATP activates inward cation currents and enhances neuronal excitability [10, 13]. In experimental pain states, P2X3R expression and membrane availability are frequently increased, making primary sensory neurons more responsive to purinergic stimulation and contributing to peripheral sensitization [14, 15, 28]. P2X3R may also interact with transient receptor potential vanilloid 1 (TRPV1) in peripheral nociceptive neurons, and EA has been reported to inhibit this P2X3R–TRPV1 interaction across different pathological pain models [29].

EA has repeatedly been shown to downregulate this P2X3R-mediated nociceptive pathway across different preclinical pain models [13, 14, 30]. Tu et al. [13] demonstrated that EA reduced ATP-evoked P2X3 currents in DRG neurons from chronic constriction injury (CCI) rats, providing functional electrophysiological evidence beyond receptor expression changes alone. Xiang et al. [14] showed that 100 Hz EA at ST36 and SP6 suppressed P2X3R expression in DRG and spinal dorsal horn tissue in complete Freund's adjuvant (CFA)-induced inflammatory pain, with parallel reductions in thermal and mechanical hyperalgesia. In diabetic neuropathic pain, Zhou et al. [15] provided a more specific mechanistic explanation by showing that EA suppresses protein kinase C (PKC)-dependent P2X3R membrane trafficking, indicating that EA reduces receptor availability at the neuronal surface.

This receptor-level pattern has been extended to neuropathic pain, diabetic neuropathy, visceral pain, and bone cancer pain models, supporting the broader relevance of P2X3R modulation in EA analgesia [28, 30–34]. EA frequency may influence purinergic modulation, but current evidence is insufficient to identify a universally optimal frequency. In diabetic neuropathic pain, He et al. [31] directly compared 2 and 100 Hz EA and reported frequency-dependent effects on hyperalgesia-related protein expression, while Zhou et al. [15] linked EA analgesia to suppression of PKC-dependent P2X3R membrane trafficking. Taken together, these findings raise the possibility that low- and high-frequency stimulation engage P2X3R-associated pathways differently. However, comparisons are constrained by differences in intensity, treatment schedule, acupoint selection, disease model, molecular outcome, and tissue-collection timing. Frequency should therefore be considered a biologically relevant protocol variable rather than evidence that one frequency is universally superior. The breadth of replication across multiple disease models nevertheless makes P2X3R the most credible peripheral purinergic receptor target associated with EA to date [3, 4, 6], although direct receptor-level confirmation in humans is still lacking [4, 11].

## P2X4 receptors: a spinal microglial bridge to central sensitization

P2X4 receptors are particularly important at the spinal level because they are closely linked to microglial activation and central sensitization in neuropathic and inflammatory pain states [16–18]. Under resting conditions, P2X4R expression in spinal microglia is relatively low, whereas peripheral nerve injury or inflammatory stimulation can activate microglia and increase P2X4R expression [16, 35]. P2X4R upregulation is associated with BDNF release, altered dorsal horn neuronal excitability, and facilitated nociceptive transmission [16, 17]. In the EA literature, these observations support a putative P2X4R-related neuroimmune pathway, but they do not by themselves establish receptor-specific causality.

Chen et al. [16] provided one of the most mechanistically informative studies linking EA with spinal P2X4R changes. In a CCI rat model, peripheral nerve injury increased interferon-gamma (IFN-γ), which was associated with P2X4R upregulation in spinal microglia; EA at ST36 reduced the IFN-γ–P2X4R signal and mechanical allodynia. Qu et al. [18] likewise reported that EA was accompanied by reduced spinal microglial P2X4R and downstream neuroinflammatory mediators, including BDNF, interleukin-1 beta (IL-1β), and tumour necrosis factor-alpha (TNF-α), in diabetic neuropathic pain. Zheng et al. [17] linked EA-associated changes in the P2X4R pathway to reduced excitatory activity in substantia gelatinosa neurons. These findings are consistent with P2X4R involvement but should be interpreted as convergent associative mechanistic evidence unless receptor-specific causal manipulation is demonstrated.

Importantly, direct receptor-specific causality remains unconfirmed, as the cited EA studies did not employ P2X4R-targeted siRNA- or shRNA-based knockdown approaches. The available evidence primarily demonstrates parallel changes in EA analgesia, microglial activation, P2X4R expression, downstream BDNF/neuroinflammatory signaling, and neuronal excitability [16–18]. The spinal P2X4R literature is therefore conceptually important and supports a putative contribution to EA analgesia, particularly in neuropathic and diabetic neuropathic pain. Future studies using cell-type-specific knockdown, knockout, or other validated receptor-specific perturbations are needed to determine whether P2X4R is necessary or sufficient for the analgesic effect of EA.

## P2X7 receptors: the central paradox of the review

P2X7R is the most conceptually complex receptor subtype in this review because its role in EA analgesia cannot be reduced to a single inhibitory or excitatory direction [19–22]. Unlike P2X3R, P2X7R is a relatively low-sensitivity ATP-gated receptor that generally requires comparatively high extracellular ATP concentrations and/or sustained ATP exposure for robust activation [2, 10]. This property is crucial when interpreting EA studies: delivery of electrical stimulation does not by itself establish that extracellular ATP reached the concentration and duration required for full P2X7R activation. P2X7R signaling may vary according to cell type, anatomical compartment, ATP dynamics, inflammatory state, and pain model [19, 21, 33, 36], and EA frequency, intensity, pulse pattern, session duration, repetition, and acupoint selection may influence those nucleotide dynamics.

At the DRG level, P2X7R is prominently expressed in satellite glial cells (SGC), where it regulates glial–neuronal communication and inflammatory signaling around primary sensory neurons [19, 22, 37]. In inflammatory pain models, EA has been associated with suppression of a satellite glial P2X7R–p38 MAPK–TNF-α pathway [22, 37]. Wu et al. [22] used EA at ST36 and BL60 with an alternating 2/100 Hz pattern, pulse widths of 0.4 ms at 2 Hz and 0.2 ms at 100 Hz, stepwise intensities of 0.5, 1.0, and 1.5 mA for 10 min each (30 min total), administered daily for seven sessions. That study also used pharmacological interventions and AAV-mediated shRNA knockdown in SGCs, providing stronger cell-targeted evidence than expression changes alone. Lv et al. [33] identified a related DRG P2X7R–P2Y1R–P2X3R cascade in IBS-like visceral hypersensitivity; where full stimulation parameters are not consistently reported in secondary descriptions, they should not be inferred.

At the spinal level, EA-associated inhibition of P2X7R appears analgesic in several models, particularly through astrocyte-dependent central sensitization [20, 38]. Weng et al. [20] applied EA at bilateral ST36 and SP6 at 2 Hz and 1 mA for 15 min per session over seven consecutive days; pharmacological P2X7R agonist/antagonist experiments supported pathway involvement in visceral hypersensitivity. Wu et al. [38] similarly linked EA with reduced spinal P2X7R expression, synaptic remodeling, and neuropathic pain behavior, although stimulation parameters and the strength of receptor-specific causal inference should be interpreted from the original protocol rather than generalized across studies. These protocol differences matter because P2X7R activation depends on extracellular ATP exposure, which was not directly quantified alongside receptor activation in most studies.

The paradox emerges at the supraspinal level, where P2X7R activation rather than inhibition may contribute to EA analgesia [21]. Zhao et al. [21] used EA at the Yongquan acupoint with alternating 2/200 Hz stimulation at 0.5 mA for 30 min and found that astrocytic P2X7R activation in the retrosplenial cortex (RSC) supported EA-induced analgesia in inflammatory pain. Suppression of cortical astrocytic P2X7R weakened the analgesic effect of EA, contrasting with inhibitory P2X7R patterns reported in peripheral satellite glial cells and spinal astrocytes [20–22]. This finding should not be forced into a simple inhibitory model: P2X7R may be pain-promoting in peripheral and spinal inflammatory glial contexts but analgesia-supportive in selected cortical networks. The marked heterogeneity in EA parameters across these studies further cautions against attributing directionality to receptor location alone.

The most scientifically balanced interpretation is therefore not that P2X7R is uniformly beneficial or harmful, but that its function is compartment-specific, context-dependent, and potentially sensitive to stimulation-dependent extracellular ATP dynamics [19, 21, 36]. Across the available studies, protocols range from 2 Hz, 1 mA, 15-min repeated sessions [20], to alternating 2/200 Hz at 0.5 mA for 30 min [21], and alternating 2/100 Hz with stepwise 0.5–1.5 mA intensities over 30 min [22]. Because most studies do not simultaneously quantify extracellular ATP concentration and P2X7R activation, it remains uncertain whether protocol-dependent ATP exposure explains part of the observed directional heterogeneity. Direct ATP measurements combined with receptor activation readouts and standardized EA reporting are therefore priorities for future work.

## Integrated mechanistic model

**Fig. 1:**
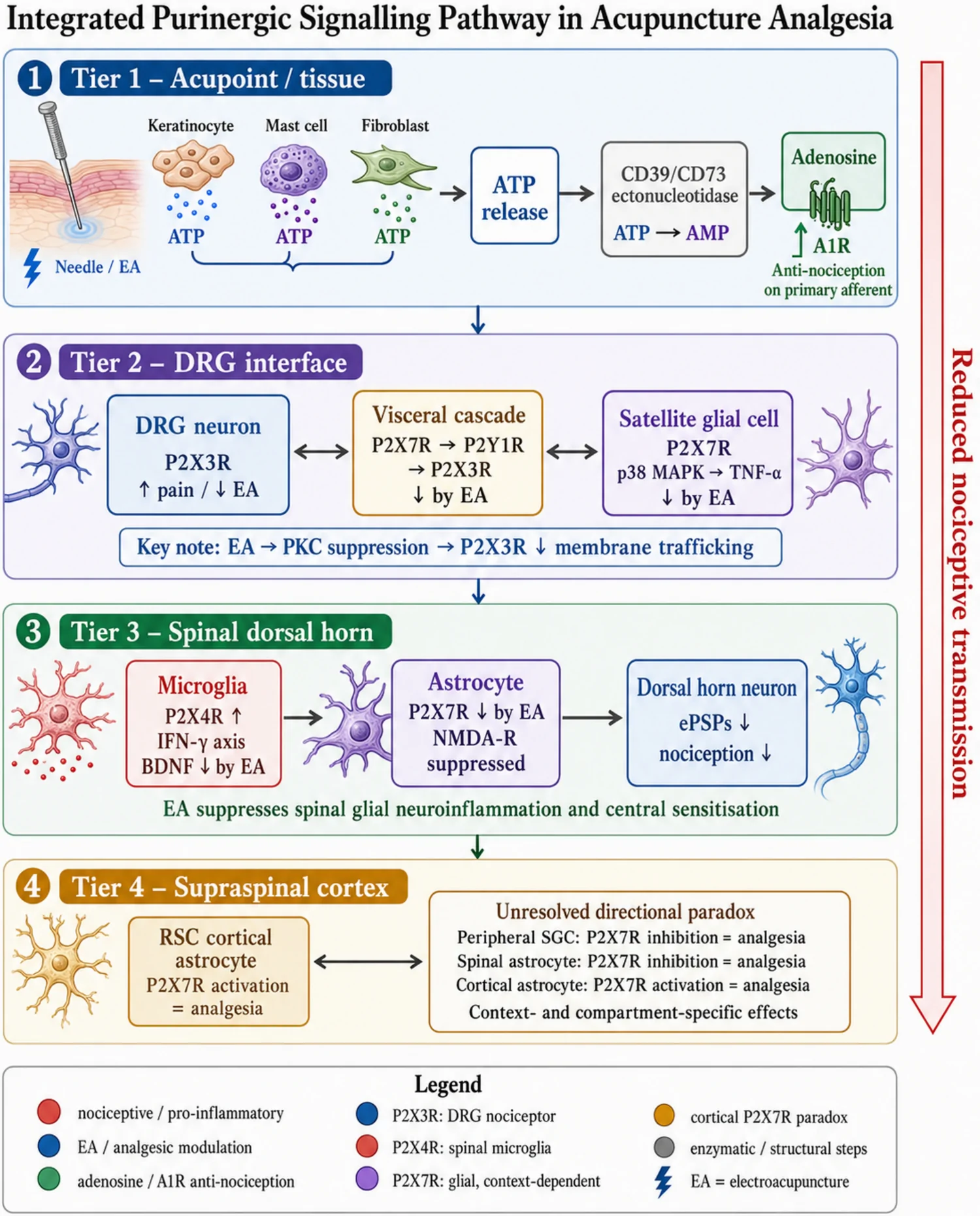
Integrated purinergic signaling mechanisms underlying needling-induced analgesia. Manual/traditional acupuncture supports the local ATP–adenosine–A1R pathway, whereas most receptor-level P2X3R, P2X4R, and P2X7R evidence derives from EA. EA is associated with reduced DRG P2X3R signaling, reduced satellite glial P2X7R inflammatory signaling, a putative reduction in spinal microglial P2X4R/BDNF signaling, and reduced spinal astrocytic P2X7R/NMDA-R sensitization. Cortical astrocytic P2X7R activation in the retrosplenial cortex may support EA analgesia, highlighting a compartment-specific directional paradox. Figure created by the author for this review

Figure 1 summarizes the proposed four-level model. The figure integrates the mechanistic argument: an acupoint ATP–adenosine trigger, peripheral P2X3R modulation, a putative spinal P2X4R-associated neuroimmune pathway, spinal P2X7R-related glial modulation, and the supraspinal P2X7R directional paradox. A single integrative figure is preferable to multiple smaller figures because the scientific value of this paper lies in connecting these mechanisms across anatomical levels while distinguishing the strength and modality of the underlying evidence.

## Evidence map by receptor subtype

The mechanistic evidence is summarized in two complementary tables. Table 1 provides a study-level overview of representative major experimental and translational studies linking manual/traditional acupuncture or EA to purinergic signaling, including pain model, stimulation method, receptor subtype or pathway, key mechanistic finding, and main limitation. Table 2 synthesizes evidence by receptor subtype and anatomical site, comparing P2X3R, P2X4R, P2X7R, and the ATP–adenosine–A1R pathway according to their proposed role in EA or needling-related analgesia and overall strength of evidence. Together, these tables show both individual study foundations and the broader receptor-level interpretation.

## Disease-model-specific interpretation

Inflammatory pain models, particularly CFA-induced inflammation, provide some of the clearest evidence for the acupoint ATP–adenosine pathway, DRG P2X3R suppression, and satellite glial P2X7R involvement in EA analgesia [5, 14, 22]. These models are valuable because they produce robust peripheral inflammation and measurable thermal or mechanical hyperalgesia, allowing receptor-level analgesic mechanisms to be experimentally tested [5, 14]. However, CFA-based animal models cannot fully reproduce the chronicity, heterogeneity, affective burden, and psychosocial complexity of human inflammatory pain conditions [1, 4].

Neuropathic pain models, including CCI and spinal nerve ligation (SNL), are especially informative for studying spinal neuroimmune mechanisms of EA analgesia [13, 16, 32, 38]. In these models, EA modulates both peripheral DRG P2X3R signaling and spinal glial receptor pathways, particularly P2X4R-mediated microglial activation and P2X7R-related spinal sensitization [13, 16, 32, 38]. This suggests that neuropathic pain may require multi-level purinergic modulation involving both peripheral nociceptive neurons and central glial mechanisms, rather than targeting a single peripheral receptor pathway alone [1, 4, 6].

Diabetic neuropathic pain models add mechanistic specificity because EA appears to suppress PKC-dependent P2X3R membrane trafficking in DRG neurons and is associated with reduced spinal microglial P2X4R-related neuroinflammation [15, 18, 30, 31]. Additional evidence suggests that DRG P2X4R and P2X7R may contribute to diabetes-induced hyperalgesia and may be downregulated by EA, indicating broader purinergic modulation beyond P2X3R alone [39]. Frequency is a potentially important modifier: comparative evidence suggests that 2 and 100 Hz EA can produce different effects on hyperalgesia-related protein expression [31], but the receptor-specific implications remain uncertain and should not be generalized beyond the tested protocols.

Visceral pain and IBS models are important because they reveal purinergic receptor cross-talk rather than isolated receptor changes [20, 33]. EA at abdominal acupoints has been linked to suppression of a DRG P2X7R–P2Y1R–P2X3R cascade and inhibition of spinal astrocytic P2X7R/NMDA-R signaling in IBS-like visceral hypersensitivity models [20, 33]. This pattern may represent a visceral-pain-specific purinergic signature, although its generalizability to other pain states remains uncertain [33, 40].

Bone cancer pain evidence is more limited but clinically relevant because acupuncture is often considered in supportive oncology care for pain-related symptom management [1, 28]. In a rat bone cancer pain model, EA reduced P2X3R expression and membrane trafficking in DRG neurons, suggesting that the P2X3R mechanism may extend to oncological pain contexts [28]. However, the roles of P2X4R and P2X7R in EA-mediated modulation of cancer-related pain remain insufficiently mapped, and receptor-level human evidence in oncology populations is still lacking [4, 28].

## Translational significance

The translational promise of this field lies in moving EA and other needling modalities from general analgesic interventions toward mechanism-informed strategies grounded in identifiable purinergic pathways [3–5]. If future human studies confirm that specific purinergic biomarkers predict or mediate treatment response, protocols could eventually be tailored according to pain phenotype, receptor pathway, acupoint selection, stimulation modality, and EA parameters [4, 6, 11]. Because the receptor-level evidence is predominantly EA-based, translation should preserve the distinction between electrical and manual stimulation rather than assuming equivalence.

The ATP–adenosine–A1R pathway is currently the closest purinergic mechanism to clinical translation because it has been demonstrated in mouse acupoint tissue and in human volunteers undergoing traditional acupuncture [5, 11]. P2X3R is the most attractive receptor-level EA-associated target because its direction of effect is relatively consistent and it is repeatedly downregulated across several preclinical pain models [13–15, 28]. P2X4R is relevant to neuropathic and diabetic neuropathic pain because EA-associated analgesia parallels reduced spinal microglial P2X4R expression, BDNF signaling, and neuroinflammation [16–18], but direct receptor-specific causality remains insufficiently established. P2X7R is not suitable for simple therapeutic generalization because inhibition appears analgesic in peripheral satellite glial cells and spinal astrocytes, whereas cortical astrocytic activation in the RSC may support EA analgesia [20–22].

A further translational strategy is to test whether the physical properties of acupuncture needles and EA electrodes can be optimized to modulate mechanotransduction, local ATP release, or current delivery. This remains a hypothesis-driven research direction rather than an established clinical approach. Experimental work has shown that surface texture can alter mechanical interaction with acupoint tissue [41], while modified needle materials and surface treatments can change electrical conductivity or resistance during EA-related applications [42, 43]. These findings justify direct studies comparing stainless steel, coated, conductive, or micro/nanotextured needles while simultaneously measuring local ATP, adenosine, CD39/CD73 activity, and analgesic outcomes. Other testable strategies include optimizing EA frequency, intensity, pulse pattern, and duration; selecting acupoints according to local tissue and ectonucleotidase characteristics; and exploring biomarker-guided personalization using ATP, adenosine, CD39/CD73, inflammatory mediators, and receptor-related markers. Pharmacological augmentation of adenosine metabolism or A1R signaling is also conceptually attractive, but any combination strategy would require careful safety testing and demonstration that it enhances analgesia without disrupting spatially patterned purinergic signaling. Overall, material engineering and protocol optimization should be evaluated as experimentally testable strategies, not as clinically proven methods for enhancing purinergic analgesia.

## Controversies and unresolved questions

Several unresolved issues limit direct translation of the acupuncture–P2X receptor literature into clinical practice. The most important is the P2X7R directional paradox. Current evidence suggests that EA-mediated inhibition of P2X7R may be analgesic in DRG satellite glial cells and spinal astrocytes, whereas activation of astrocytic P2X7R in the RSC may support EA analgesia [20–22]. This indicates that P2X7R cannot be interpreted as uniformly pro-nociceptive or anti-nociceptive; instead, its function appears to depend on anatomical compartment, glial cell type, inflammatory context, and pain phenotype.

A second unresolved issue is the difference between manual acupuncture and electroacupuncture. The ATP–adenosine–A1R pathway was demonstrated prominently using manual needle stimulation, and human microdialysis evidence comes from traditional acupuncture at ST36 [5, 11]. In contrast, most receptor-level studies of P2X3R, P2X4R, and P2X7R rely on EA protocols. This modality imbalance is scientifically important because the molecular effects of manual acupuncture and EA may overlap but should not be assumed to be identical, creating an important translational gap.

Third, sex differences remain insufficiently addressed. Many mechanistic studies use rodent models with limited or unclear sex-balanced reporting, even though chronic pain is biologically heterogeneous and clinically variable across patient populations [1, 4]. Future work should determine whether EA-induced modulation of P2X3R, P2X4R, and P2X7R differs between males and females.

Fourth, EA frequency dependence remains incompletely resolved. Available comparative evidence suggests that EA frequency may influence P2X3R-associated mechanisms [15, 31], although interpretation remains constrained by differences in intensity, pulse pattern, acupoint selection, treatment duration, repetition, pain model, and tissue-collection timing. For P2X7R in particular, such parameters may influence extracellular ATP dynamics, yet direct ATP measurements are rarely paired with receptor activation readouts.

Finally, human translation remains the central challenge. The ATP–adenosine pathway has been demonstrated in humans, but direct human evidence for acupuncture-induced modulation of P2X3R, P2X4R, or P2X7R is still lacking [4, 6, 11]. The field therefore needs clinical studies that combine pain outcomes with mechanistic biomarkers, rather than relying only on animal receptor data or clinical symptom scores.

## Limitations of the evidence base

The first limitation is species translation. Most mechanistic evidence comes from rats and mice, whereas human receptor-level data are absent. Human acupoint adenosine accumulation supports the upstream cascade, but no human study has yet measured P2X3R, P2X4R, or P2X7R expression or function after acupuncture.

The second limitation is sex bias. Many animal studies use male rodents, limiting generalizability to clinical pain populations in which women are often disproportionately affected. Future studies should include sex-balanced designs and report sex-stratified outcomes.

The third limitation is modality bias. The receptor-level mechanistic literature is dominated by EA, while manual acupuncture is underrepresented. This matters because manual needle rotation was central to the original ATP–adenosine findings, whereas most P2X3R, P2X4R, and P2X7R studies use electrical stimulation with variable parameters. The two modalities should not be treated as mechanistically interchangeable.

The fourth limitation is methodological heterogeneity. Studies vary in acupoints, stimulation frequency, intensity, pulse pattern, duration, repetition, pain model, receptor assay, tissue collection timing, and behavioural endpoint. These differences make quantitative pooling inappropriate and justify the narrative evidence-mapping design used here. For P2X7R, the lack of simultaneous measurement of extracellular ATP concentration and receptor activation is an additional mechanistic limitation.

The fifth limitation is conceptual: P2X7R directionality remains unresolved. The field needs a framework that explains when P2X7R activation is pro-nociceptive, when inhibition is analgesic, and why cortical activation may support analgesia.

## Future directions

Future research should move beyond descriptive receptor profiling toward human mechanistic studies that test whether purinergic changes mediate acupuncture-related pain relief. Although the ATP–adenosine–A1R pathway has been demonstrated in both animal and human acupoint studies, direct human evidence for acupuncture-induced modulation of P2X3R, P2X4R, or P2X7R remains lacking [4–6, 11]. Future microdialysis studies could therefore expand beyond adenosine to include ATP, AMP, CD39/CD73 activity, and local inflammatory mediators, particularly because CD39/CD73-mediated ATP–adenosine conversion appears central to acupuncture analgesia [9, 12].

Preclinical studies should adopt sex-balanced designs and directly compare EA frequencies and other stimulation parameters within the same experimental models. Frequency-dependent effects have been reported in diabetic neuropathic pain and P2X3R-related mechanisms [15, 31], while the P2X7R literature spans markedly different frequency, intensity, and treatment schedules [20–22]. Standardized reporting of acupoints, needle characteristics, frequency, intensity, pulse width/pattern, duration, repetition, tissue collection timing, and receptor assays would improve comparability. Studies should also pair extracellular ATP measurements with receptor activation readouts when testing P2X7R-related mechanisms.

Resolving the P2X7R paradox should be a major priority. Current evidence suggests that P2X7R inhibition may contribute to analgesia in DRG satellite glial cells and spinal astrocytes, whereas P2X7R activation in RSC astrocytes may support EA analgesia [20–22]. Future studies should use cell-type-specific manipulation across comparable pain models to determine whether anatomical compartment, cell type, ATP concentration, or disease state determines P2X7R directionality.

Finally, clinical trials should incorporate mechanistic substudies so that biomarker changes can be linked to treatment response. The next stage of the field should ask not only whether acupuncture or EA reduces pain, but which purinergic pathways change in responders and whether those changes explain clinical benefit [3, 4, 6]. Comparative studies of manual acupuncture and EA, together with experimentally controlled needle materials and surface properties, may help determine which aspects of mechanical and electrical stimulation are responsible for local ATP mobilization and downstream analgesia.

## Conclusion

P2X purinergic receptors provide a compelling mechanistic framework for EA-related analgesia, situated within a broader needling-induced purinergic model. The model begins locally: manual/traditional needling promotes ATP release at acupoints, ATP is converted through CD39/CD73 into adenosine, and adenosine activates A1R to produce anti-nociception. Downstream receptor-level evidence derives predominantly from EA. EA appears to reduce peripheral sensitization primarily through P2X3R downregulation in DRG nociceptive neurons. At the spinal level, EA analgesia is associated with reduced microglial P2X4R/BDNF signaling, although direct receptor-specific causality remains limited, and with modulation of astrocytic P2X7R/NMDA-R pathways. At the supraspinal level, P2X7R becomes more complex because cortical astrocytic activation may support analgesia rather than oppose it.

The strongest conclusion is therefore balanced: purinergic signaling is a biologically plausible and increasingly supported bridge between needling-based stimulation and pain relief, but the framework remains incompletely validated in humans and is dominated by EA at the receptor level. P2X3R is currently the most consistent EA-associated receptor target, P2X4R provides a coherent but not yet definitively causal spinal neuroimmune hypothesis, and P2X7R defines the most important compartment- and protocol-sensitive unresolved question. Future progress depends on human mechanistic studies, sex-balanced designs, standardized EA parameters, direct extracellular ATP measurements, cell-type-specific receptor interrogation, and explicit distinction between manual acupuncture and EA.

## Data Availability

No datasets were generated or analysed during the current study.

## Declarations

### AI-use statement

AI-assisted editing was used only for language polishing, formatting support, and readability improvement. The author reviewed, verified, and approved all content and remains fully responsible for the final manuscript.

## Abbreviations

*A1R*: Adenosine A1 Receptor
*AAV*: Adeno-Associated Virus
*ATP*: Adenosine Triphosphate
*BDNF*: Brain-Derived Neurotrophic Factor
*CD39*: Ectonucleoside Triphosphate Diphosphohydrolase-1
*CD73*: Ecto-5′-Nucleotidase
*CCI*: Chronic Constriction Injury
*CFA*: Complete Freund’s Adjuvant
*DRG*: Dorsal Root Ganglion
*EA*: Electroacupuncture
*IFN*-γ: Interferon-Gamma
*IBS*: Irritable Bowel Syndrome
*IL-1β*: Interleukin-1 Beta
*MAPK*: Mitogen-Activated Protein Kinase
*NMDA*: N-Methyl-D-Aspartate
*NMDA-R*: N-Methyl-D-Aspartate Receptor
*P2X3R*: P2X3 Receptor
*P2X4R*: P2X4 Receptor
*P2X7R*: P2X7 Receptor
*P2Y1R*: P2Y1 Receptor
*PKC*: Protein Kinase C
*RSC*: Retrosplenial Cortex
*SGC*: Satellite Glial Cell
*TNF-α*: Tumour Necrosis Factor-Alpha
*TRPV1*: Transient Receptor Potential Vanilloid 1
*TRPV4*: Transient Receptor Potential Vanilloid 4
